# Deregulation of methylation of transcribed-ultra conserved regions in colorectal cancer and their value for detection of adenomas and adenocarcinomas

**DOI:** 10.18632/oncotarget.25115

**Published:** 2018-04-20

**Authors:** Anastasia E. Kottorou, Anna G. Antonacopoulou, Foteinos-Ioannis D. Dimitrakopoulos, Georgia Diamantopoulou, Chaido Sirinian, Melpomeni Kalofonou, Theodoros Theodorakopoulos, Chrysa Oikonomou, Evangelos C. Katsakoulis, Angelos Koutras, Thomas Makatsoris, Nikos Demopoulos, Georgia Stephanou, Michalis Stavropoulos, Konstantinos C. Thomopoulos, Haralabos P. Kalofonos

**Affiliations:** ^1^ Clinical and Molecular Oncology Laboratory, Division of Oncology, Medical School, University of Patras, Patras, Greece; ^2^ Division of Gastroenterology, University Hospital of Patras, Patras, Greece; ^3^ Division of Oncology, University Hospital of Patras, Patras, Greece; ^4^ Division of Genetics, Cell and Developmental Biology, Department of Biology, University of Patras, Patras, Greece; ^5^ Department of Surgery, Medical School, University of Patras, Patras, Greece; ^6^ Institute of Biomedical Engineering, Imperial College London, London, UK

**Keywords:** transcribed-ultra conserved regions, colorectal cancer and adenomas, tissue and plasma methylation, screening biomarker, expression levels

## Abstract

Expression of Transcribed Ultraconserved Regions (T-UCRs) is often deregulated in cancer. The present study assesses the expression and methylation of three T-UCRs (Uc160, Uc283 and Uc346) in colorectal cancer (CRC) and explores the potential of T-UCR methylation in circulating DNA for the detection of adenomas and adenocarcinomas.

Expression levels of Uc160, Uc283 and Uc346 were lower in neoplastic tissues from 64 CRC patients (statistically significant for Uc160, *p*<0.001), compared to non-malignant tissues, while methylation levels displayed the inverse pattern (*p*<0.001, *p*=0.001 and *p*=0.004 respectively). In colon cancer cell lines, overexpression of Uc160 and Uc346 led to increased proliferation and migration rates. Methylation levels of Uc160 in plasma of 50 CRC, 59 adenoma patients, 40 healthy subjects and 12 patients with colon inflammation or diverticulosis predicted the presence of CRC with 35% sensitivity and 89% specificity (*p*=0.016), while methylation levels of the combination of all three T-UCRs resulted in 45% sensitivity and 74.3% specificity (*p*=0.013). In conclusion, studied T-UCRs’ expression and methylation status are deregulated in CRC while Uc160 and Uc346 appear to have a complicated role in CRC progression. Moreover their methylation status appears a promising non-invasive screening test for CRC, provided that the sensitivity of the assay is improved.

## INTRODUCTION

Colorectal cancer (CRC) is the second leading cause of cancer-related deaths for men and third for women worldwide, with 50,260 cases estimated for 2017 in United States [1]. A long-term decline in CRC incidence rates has been noted since the mid-1980s, which has been attributed mostly to early screening through colonoscopy and removal of precancerous lesions [2, 3]. However, screening is not routinely performed for persons younger than 50 years, resulting in increased incidence rates by 2% per year from 1993 through 2013 in this age group [1]. Furthermore, it has been established that early detection of malignant bowel lesions can significantly improve survival, but many CRCs are diagnosed at later stages, when the disease becomes symptomatic [4].

Screening for CRC through analysis of genetic or epigenetic markers in circulating cell-free DNA (ccfDNA), isolated from plasma or serum specimens, can provide a minimally invasive and low-cost method with higher probability of patient adherence compared to colonoscopy. CcfDNA originates from apoptotic and necrotic cells [5, 6] and is found at higher levels in cancer patients compared to healthy individuals [7, 8]. An increasing number of studies have investigated the role of ccfDNA as a cancer biomarker, identifying tumour-derived genetic and epigenetic characteristics in serum/plasma samples [9–18]. Especially for CRC, identification of DNA mutations or methylated genetic regions, offers a clinically useful biomarker for CRC screening and monitoring response to therapy [15, 19–24].

Ultraconserved regions (UCRs) were first identified in 2004 as 481 segments longer than 200 bp, absolutely conserved between orthologous regions of the human, rat, and mouse genomes, that may have played an important role in shaping the landscape of gene regulation during mammalian evolution [25, 26]. Most UCRs are noncoding and are under negative selection that is much stronger than that in protein coding genes [27]. Specific groups of UCRs are differentially expressed or methylated in various tumor types and have been associated with disease outcome [28–40]. The majority of UCRs are transcribed (T-UCRs), while their transcription is in part regulated by methylation. In particular, Uc160, Uc283 and Uc346 in CRC cells have been found to undergo specific CpG island hypermethylation-associated silencing compared with normal tissues, while DNA hypomethylation reversed this effect [40].

The aim of this study was to compare the expression and methylation levels of Uc160, Uc283 and Uc346 in colorectal adenocarcinomas, adenomas and non-malignant colonic tissues, explore their role in CRC progression and investigate the use of their methylation status in circulating DNA as a biomarker for CRC and colorectal adenomas.

## RESULTS

### Tissue T-UCR expression

**Figure 1:**
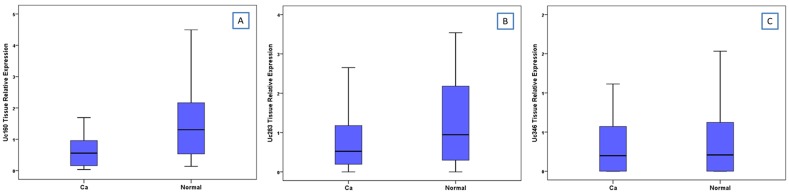
Relative expression of Uc160 **(A)**, Uc283 **(B)** and Uc346 **(C)** in adenocarcinomas and non-malignant tumor adjacent tissues.

**Figure 2:**
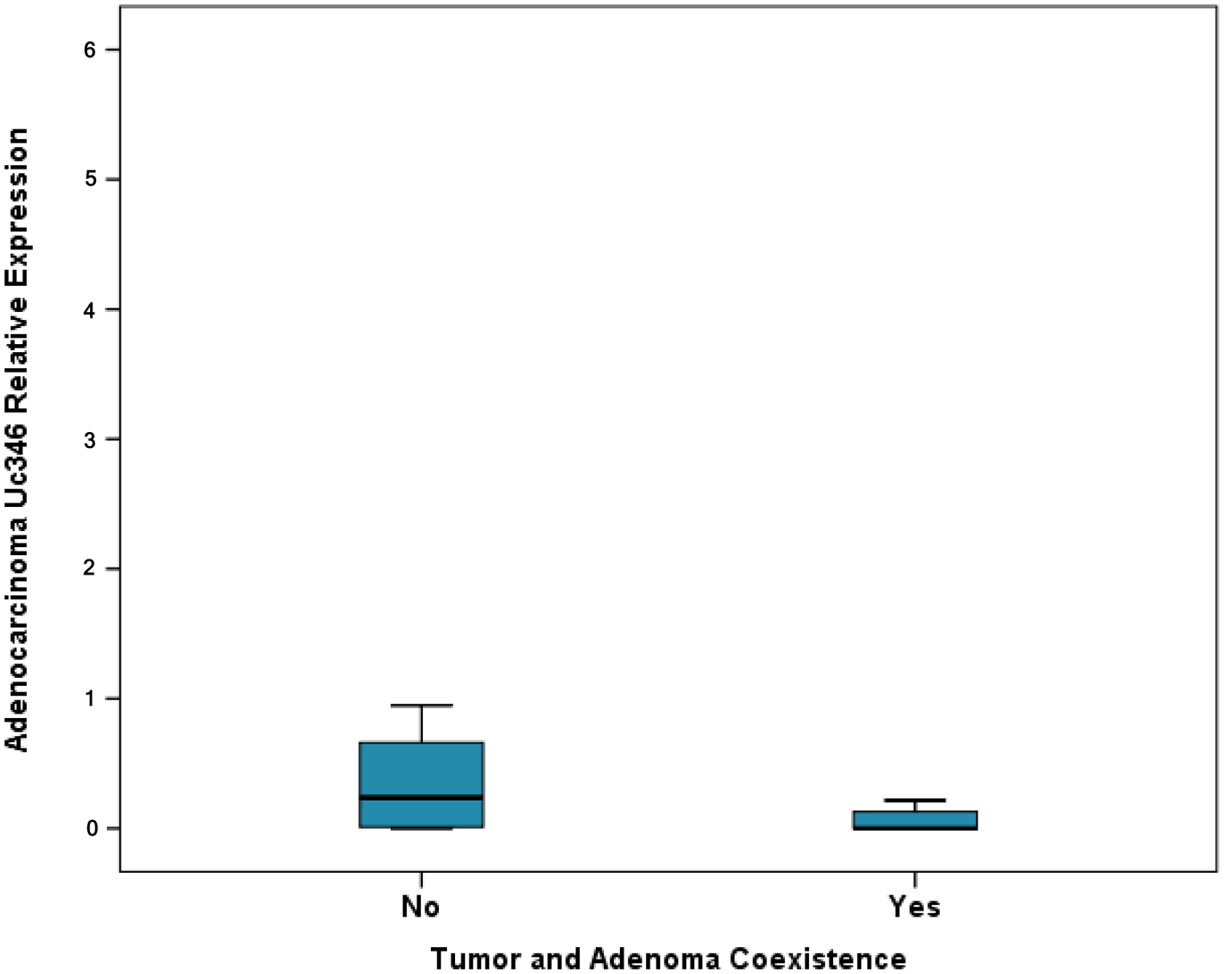
Relative expression of Uc346 in adenocarcinomas and adenocarcinoma and adenoma coexistence

Expression levels of Uc160, Uc283 and Uc346 were assessed in 51 adenocarcinomas and paired non-malignant tumor adjacent tissues. Additionally, Uc160 expression levels were assessed in 2 fresh frozen (FF) adenoma tissue specimens. Differences in expression levels were noted among adenocarcinomas and non-malignant tumor adjacent tissues. Non-malignant tissues demonstrated higher expression levels of Uc160, Uc283 and Uc346 compared to adenocarcinomas, although the difference was statistically significant only for Uc160 (*p*<0.001, *p*=0.182 and *p*=0.639 respectively, Table 1, Figure 1). T-UCRs expression levels in adenocarcinomas were not associated with sex, age, tumor type (ulcerative or exophytic), location (left or right colon or rectum), stage, lymph node infiltration, differentiation and distant metastasis (Supplementary Table 1). Similarly, T-UCR levels were also independent of lifestyle factors i.e. alcohol, meat or coffee consumption, smoking and exercise. However, lower levels of tumor Uc346 (*p*=0.011, Figure 2) was noted in patients who at the time of surgery presented with adenocarcinomas and adenomas concomitantly.

**Table 1:** Relative expression of T-UCRs in adenocarcinomas, non-malignant tumor adjacent tissues and adenomas

Tissue	Number of samples	Uc160 relative expression	*P* value	Uc283 relative expression	*P* value	Uc346 relative expression	*P* value
Non-malignant tumor adjacent	51	1.30 (0.14-4.50)	<0.001	0.94 (0-16.03)	0.182	0.21 (0-6.14)	0.639
Adenocarcinoma	51	0.56 (0.14-3.51)		0.52 (0-15.63)		0.20 (0-5.86)	
Adenoma	2	0.36 (0.15-0.57)		N/A		N/A	

### Tissue T-UCR methylation

**Figure 3:**
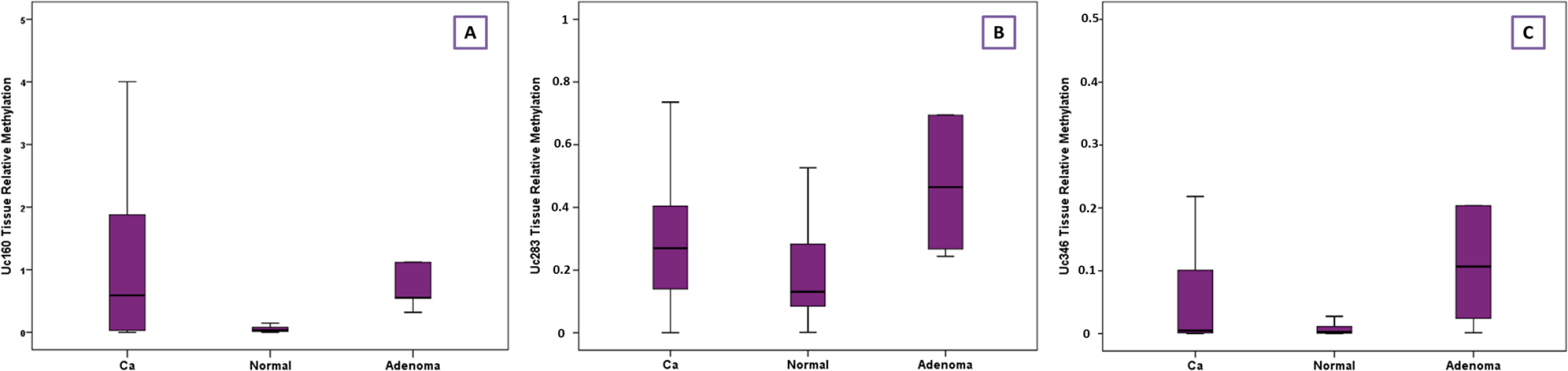
Relative methylation of Uc160 **(A)**, Uc283 **(B)** and Uc346 **(C)** in adenocarcinomas, non-malignant tumor adjacent tissues and adenomas.

**Figure 4:**
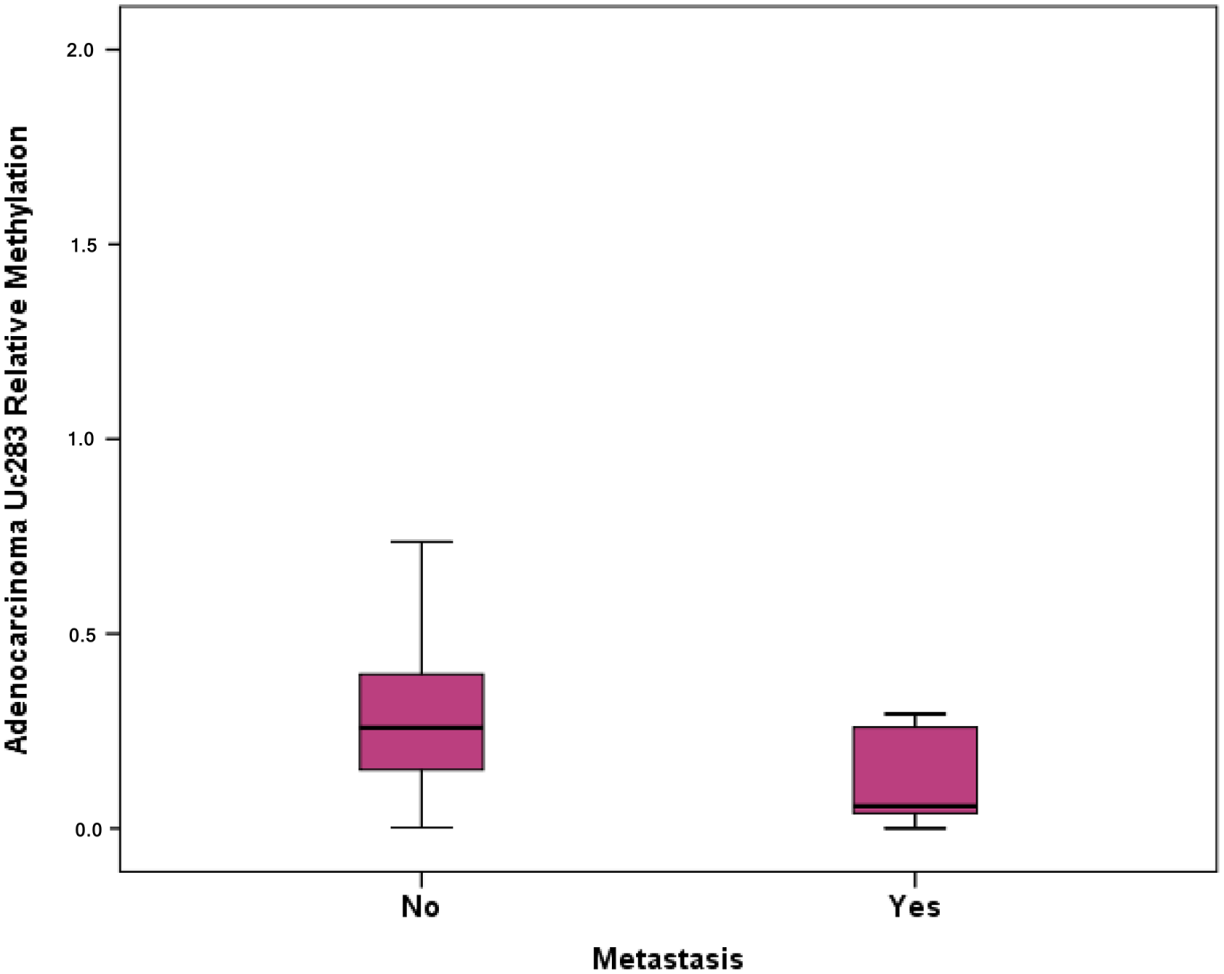
Relative methylation of Uc283 in adenocarcinomas and association with distant metastasis

Methylation levels of Uc160, Uc283 and Uc346 were assessed in 64 adenocarcinomas and paired, non-malignant, tumor-adjacent tissues, as well as in 6 FF adenomas. Methylation levels of Uc160, Uc283 and Uc346 were significantly different among adenocarcinomas, non-malignant tumor adjacent tissues and adenomas (*p*<0.001, *p*=0.001 and *p*=0.004 respectively, Table 2, Figure 3). Interestingly, the highest Uc160 methylation levels were noted in adenocarcinomas while Uc283 and Uc346 methylation levels reached the highest values in adenomas. T-UCR methylation levels in adenocarcinomas were not correlated with expression levels in both adenocarcinomas and non-malignant tumor-adjacent tissues. Moreover, they were not associated with most clinicopathological characteristics (Supplementary Table 2) or lifestyle characteristics (Data not shown). However, methylation of Uc283 in adenocarcinomas was higher in patients without distant metastasis compared to patients with distant metastasis (*p*=0.021, Figure 4). Furthermore, a trend was noted for decreasing levels of Uc283 methylation in grade II compared to grade I tumors. Notably, grade I tumors exhibited almost double median value compared to grade II tumors (*p*=0.052).

**Table 2:** Relative methylation of T-UCRs in adenocarcinomas, non-malignant tumor adjacent tissues and adenomas

Tissue	Number of samples	Uc160 relative methylation	*P* value	Uc283 relative methylation	*P* value	Uc346 relative methylation	*P* value
Non-malignant tumor adjacent	64	0.03 (0-6.22)	<0.001	0.13 (0-1.08)	0.001	0.002 (0-0.93)	0.004
Adenocarcinoma	64	0.59 (0-11.0)		0.27 (0-1.85)		0.005 (0-1.80)	
Adenoma	6	0.55 (0.31-7.82)		0.46 (0.24-2.34)		0.11 (0-1.82)	

### Plasma T-UCR methylation

T-UCR methylation levels were assessed in plasma samples from 40 healthy participants, 12 participants with colon inflammation, 50 CRC patients and 59 adenoma patients. Plasma samples from 11 CRC patients and 1 adenoma patient were collected postoperatively and were excluded from further analysis. Notably, β-actin plasma levels were higher in cancer patients compared to healthy controls, adenoma patients and inflammation patients (*p*=0.042, median values 0.20, 0.12, 0.06 and 0.04 respectively). Moreover, they were double in non-smokers compared to smokers (*p*=0.046) but were not associated with other lifestyle factors (alcohol, meat and coffee consumption and exercise), patients’ BMI, tumor or adenoma size, adenoma location, grade of dysplasia or type. The plasma T-UCR methylation frequency was the highest in CRC patients and lowest in healthy controls (Table 3). Plasma Uc160 and Uc346 methylation levels differed significantly among the four groups of participants (*p*<0.001 and *p*=0.039 respectively). More specifically, cancer patients had the highest mean values respectively (0.08 and 0.04) compared to adenoma patients (0.02 and 0.02), inflammation patients (0.04 and 0) and healthy controls (0.005 and 0.001).

**Table 3:** Frequency of plasma T-UCR methylation in different groups of participants (preoperative samples only)

Methylation in Plasma	Methylation status	Category				Total
**Healthy controls**	**Adenoma Patients**	**Ca Patients**	**Other Patients**
Uc160	Unmethylated	38 (95%)	52 (89.7%)	26 (66.7%)	9 (75%)	125
	Methylated	2 (5%)	6 (10.3%)	13 (33.3%)	3 (25%)	24
Uc283	Unmethylated	38 (95%)	53 (91.4%)	34 (87.2%)	10 (83.3%)	135
	Methylated	2 (5%)	5 (8.6%)	5 (12.8%)	2 (16.7%)	14
Uc346	Unmethylated	37 (92.5)	50 (86.2%)	30 (76.9%)	12 (100%)	129
	Methylated	3 (7.5%)	8 (13.8%)	9 (23.1%)	0 (0%)	20
Total		40	58	39	12	149

Other patients: patients with inflammation or diverticulosis

**Figure 5:**
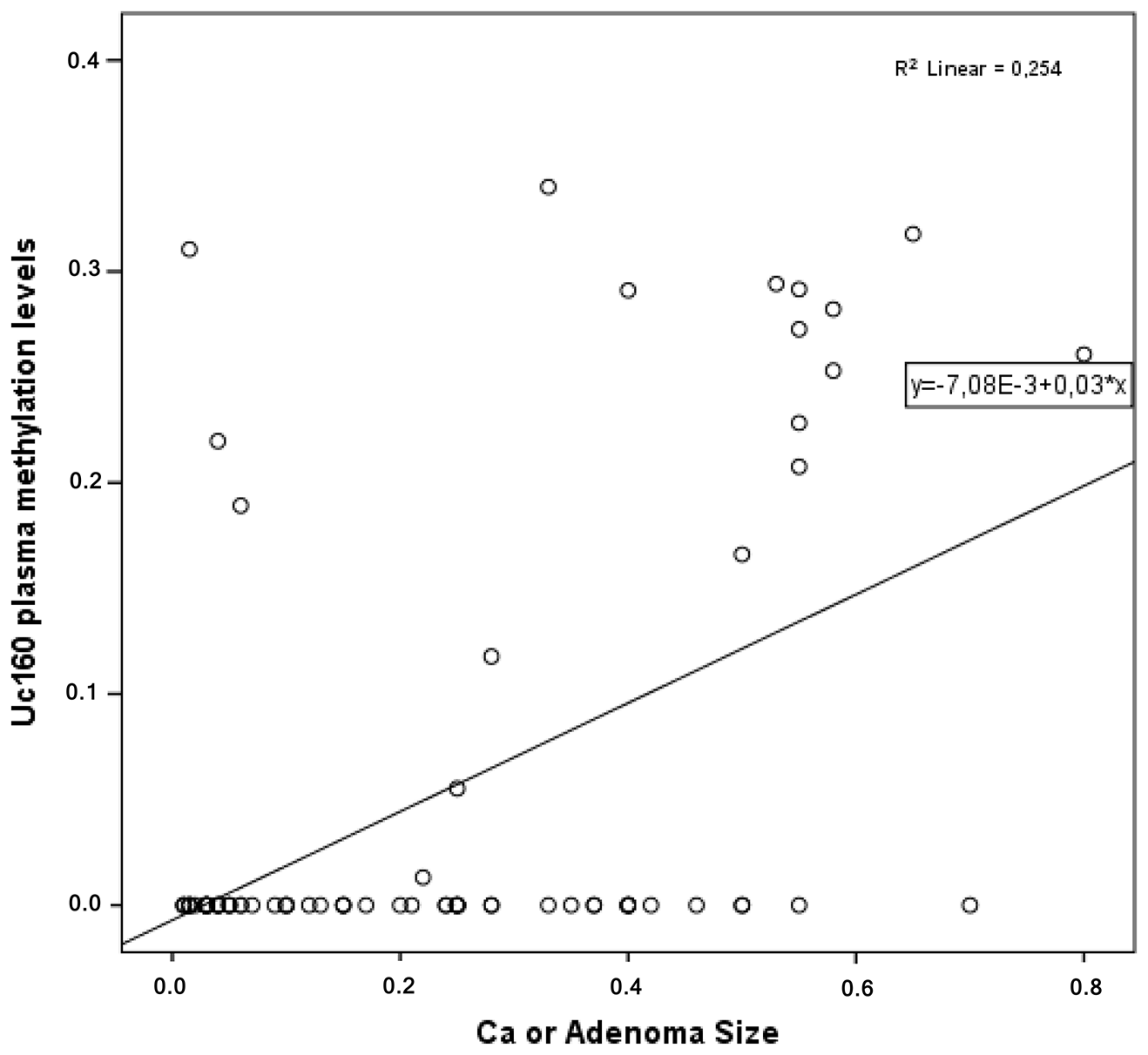
Correlation of plasma Uc160 methylation levels with adenocarcinoma or adenoma size

**Figure 6:**
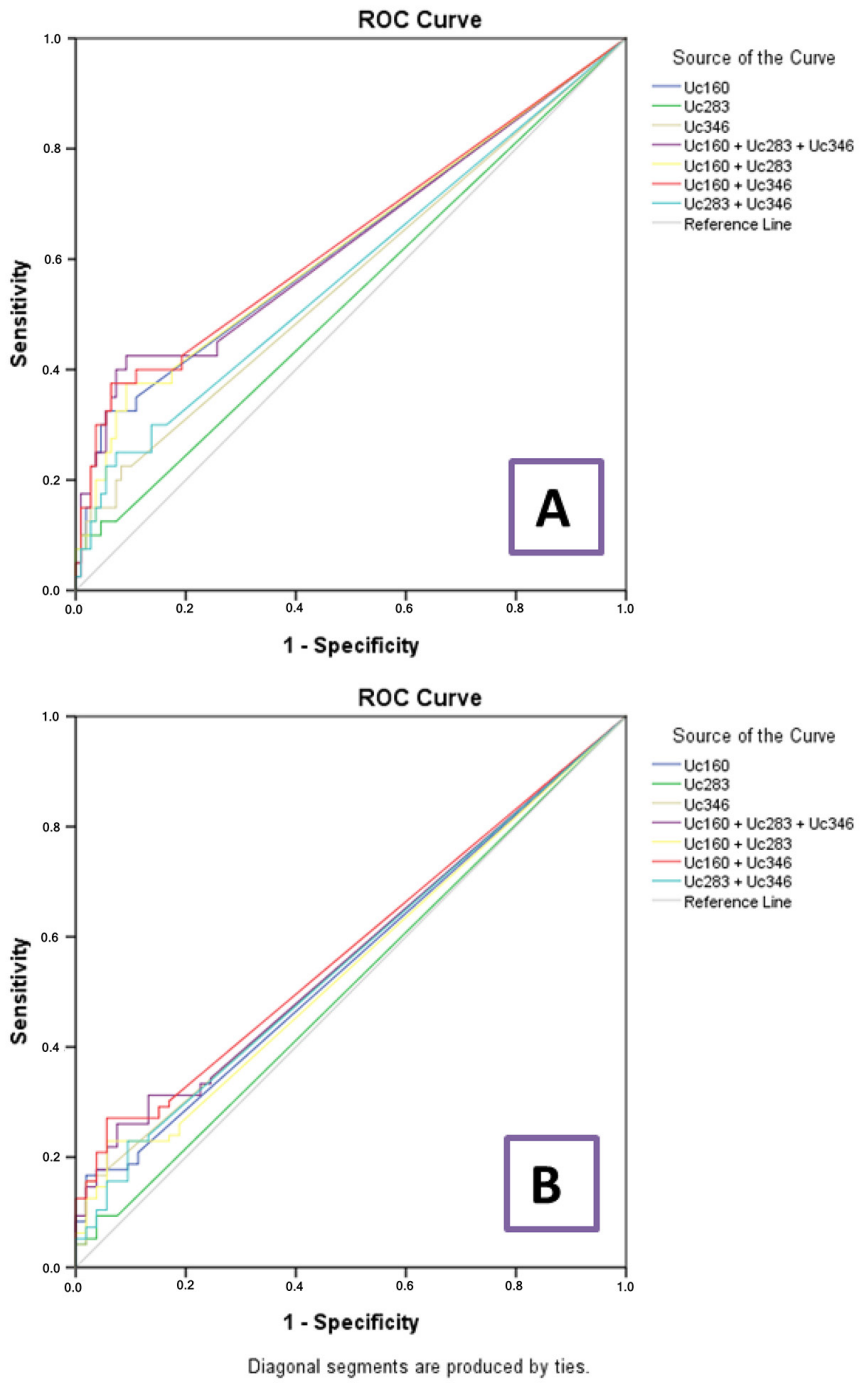
ROC curve analyses for plasma T-UCR methylation levels and colorectal adenocarcinomas **(A)** and colorectal adenocarcinomas or adenomas **(B)**.

In cancer and adenoma patients, Uc160 plasma methylation levels were positively correlated with lesion size (tumor or adenoma) (*p*<0.001, Figure 5). Moreover they were considerably higher in patients with lymph node infiltration (*p*=0.024). In addition, the frequency of methylated Uc160 in plasma was higher in male patients and in patients over 60 years (*p*=0.023 and *p*=0.040) (Table 4). With regard to Uc346 plasma methylation levels, these were correlated with tissue methylation levels (*p*=0.011). For all T-UCRs, plasma methylation levels were not associated with the life style and clinicopathological parameters mentioned above. Combinations of the three methylated T-UCRs in plasma were investigated to identify one with the highest sensitivity and specificity for adenocarcinomas (Table 5 and Figure 6A) and for adenocarcinomas or adenomas (Table 6 and Figure 6B). When taking into consideration both specificity and sensitivity, Uc160 and the sum of the three T-UCRs seem to be the most promising biomarkers for CRC, with sensitivity and specificity 35% and 89%, and 45% and 74.3% respectively (*p*=0.016 and *p*=0.013). With regard to the detection of adenomas or adenocarcinomas, Uc160 and Uc346 had 30.2% sensitivity and 80.7% specificity although statistical significance was not achieved possibly due to the small number of patients (*p*=0.160).

**Table 4:** Characteristics of the patients with positive plasma Uc160 methylation

Patient code	Sex	Age	Category	Tumor Differentiation	Lymph Node infiltration	Stage (Duke’s)	Tumor/ Adenoma Size	Tumor/Adenoma Position
045	M	64	Ad				0.5	Left colon
049	M	80	Ad				2.5	Rectum
053	F	59	CRC	Intermediate	Yes	C	5.5	Right colon
062	F	67	CRC	Intermediate	Yes	D	4.5	Left colon
064	F	49	H					
066	M	72	CRC	Low	No	A	2.8	Left Colon
077	M	40	In					
079	F	86	CRC		No	B	5.5	Right colon
084	M	61	Ad				4	Right colon
090	M	73	Ad				0.6	Right colon
095	M	80	CRC	Intermediate	Yes	D	5.8	Right colon
102	M	75	Div					
104	M	63	Ad				0.15	Left colon
113	F	83	Ad				0.4	Left colon and rectum
130	M	63	In					
131	M	77	CRC	Intermediate	No	B	5.3	Rectum
138	M	82	CRC		Yes	C	8	Rectum
147	M	64	CRC	Intermediate	Yes	C	3.3	Left colon
148	M	9	CRC	Intermediate	Yes	C	2.5	Right colon
156	M	43	CRC	Intermediate	Yes	D	5.5	Rectum
165	M	64	CRC	Intermediate	Yes	C	6.5	Left colon
198	M	80	CRC	Intermediate	Yes	C	5	Rectum
224	F	66	H					
229	M	62	CRC	Intermediate	Yes	D	5.5	Left colon
230	F	73	CRC	Intermediate	Yes	D	5.5	Rectum
243	M	69	CRC	Intermediate	No	B	5.8	Right colon

M: male, F: female, Ad: adenoma, H: healthy, In: inflammation, Div: diverticulosis

**Table 5:** Sensitivity and specificity values of plasma T-UCR methylation for colorectal adenocarcinoma

Plasma methylation levels	Sensitivity	Specificity	AUC	*P* value
Uc160	35%	89%	0.628	0.016
Uc283	12.5%	92.7%	0.529	0.591
Uc346	22.5%	77.5%	0.566	0.221
Uc160 + Uc283	40%	82.6%	0.627	0.018
Uc160 + Uc346	42.5%	80.7%	0.639	0.009
Uc283 + Uc346	30%	83.5%	0.577	0.151
Uc160 + Uc283 + Uc346	45%	74.3%	0.633	0.013

**Table 6:** Sensitivity and specificity values of plasma T-UCR methylation for colorectal adenoma or adenocarcinoma

Plasma methylation levels	Sensitivity	Specificity	AUC	*P* value
Uc160	20.8	88.7%	0.554	0.276
Uc283	9.4%	92.5%	0.511	0.826
Uc346	17.1%	94.3%	0.562	0.210
Uc160 + Uc283	26%	81.1%	0.557	0.252
Uc160 + Uc346	30.2%	83%	0.570	0.160
Uc283 + Uc346	24%	86.8%	0.548	0.332
Uc160 + Uc283 + Uc346	34.4%	75.5%	0.581	0.252

It is worth mentioning that all CRC patients whose samples were collected postoperatively had negative plasma Uc160 methylation. Exceptions were two patients who had postoperative active disease and their Uc160 levels remained positive: a stage D CRC patient (#062), who did not undergo metastasectomy, and an initially characterized as inflammation patient (#077), who was found to harbor liver metastasis from renal cancer. On the contrary, patient #134, who had a family history of CRC, maintained negative Uc160 plasma methylation status in three different time points (before undergoing colonoscopy, preoperatively and postoperatively). Further investigation into the association between the methylation status of these T-UCRs in plasma and family history of cancer revealed that a greater percentage of sporadic CRC patients presented with methylated Uc160 (57.1%) and Uc346 (25%), compared to CRC patients with a first degree relative with any type of cancer (Uc160 15.4% and Uc346 7.7%) (*p*=0.052 and *p*=0.015, respectively). This difference in Uc160 methylation status remained the same when patients with adenomas (25% in general population *vs* 7.9% in patients with first degree relatives with cancer) were included in the CRC cohort (*p*=0.050). Additionally, the methylation levels of Uc160 were higher in sporadic CRC and adenoma patients (*p*= 0.049) compared to CRC patients with a first degree relative with cancer.

### Cell proliferation

**Figure 7:**
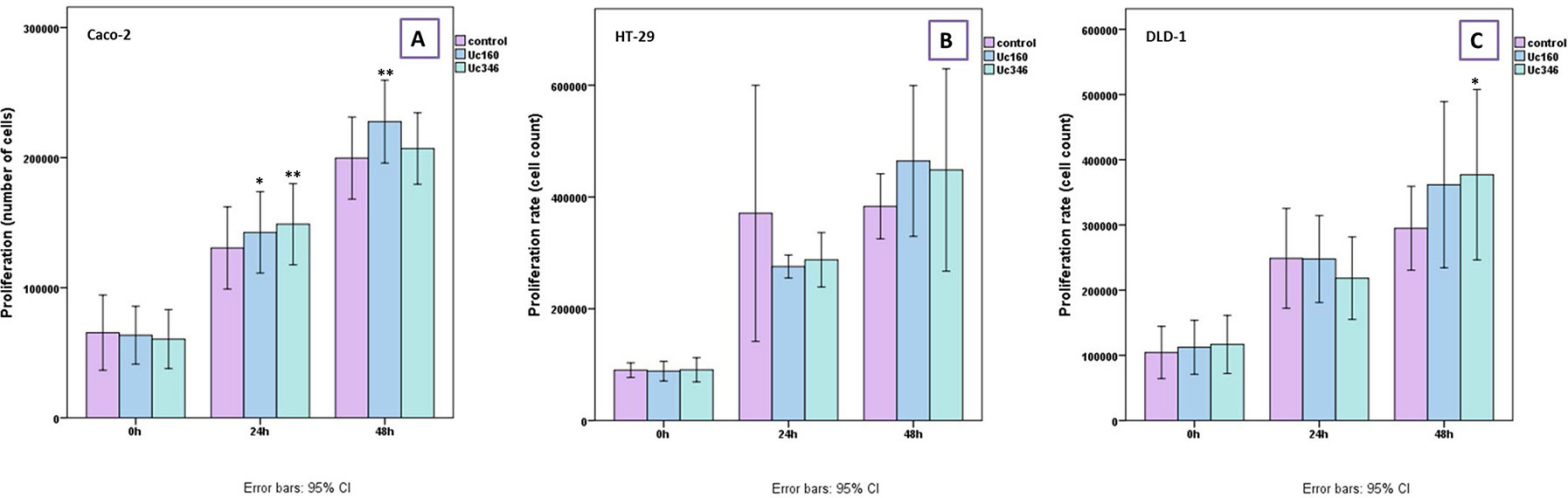
MTT proliferation assay for Caco-2 **(A)** HT-29 **(B)**, and DLD-1 **(C)** transiently transfected cells with Uc160 and Uc346 expressed in percentage of control cells (mean ±SEM).

The role of Uc160 and Uc346, which appeared to be the most promising biomarkers, was further evaluated by overexpressing these two T-UCRs in HT-29, Caco-2 and DLD-1 colon cancer cell lines. Cell proliferation rate was higher at 48h post transfection in all cell lines transfected with Uc160 and Uc346 compared to the control (Figure 7), reaching statistical significance for Uc160 in Caco-2 (*p*=0.008) and for Uc346 in DLD-1 cells (*p*=0.033).

### Cell migration

**Figure 8:**
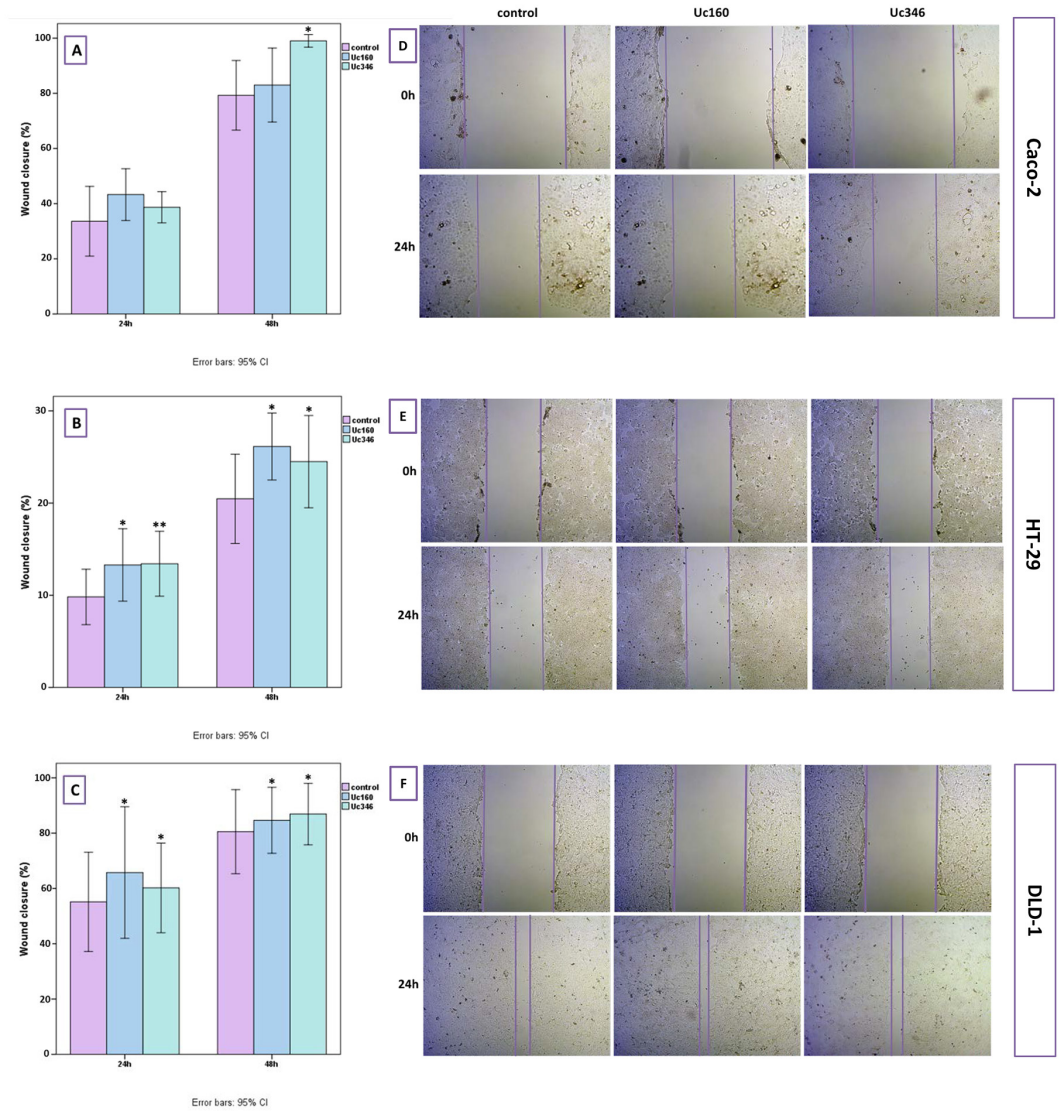
Scratch wound healing assay in HT-29, Caco-2 and DLD-1 cells transiently transfected with Uc160 and Uc346 Wound closure in Caco-2 **(A)**, HT-29 **(B)** and DLD-1 **(C)** cells in 24h and 48h as expressed in percentage of the surface at 0h (mean ±SEM). Representative images of Caco-2 **(D)**, HT-29 **(E)** and DLD-1 **(F)** cells at 0h and 24h at a magnification of 5x.

**Figure 9:**
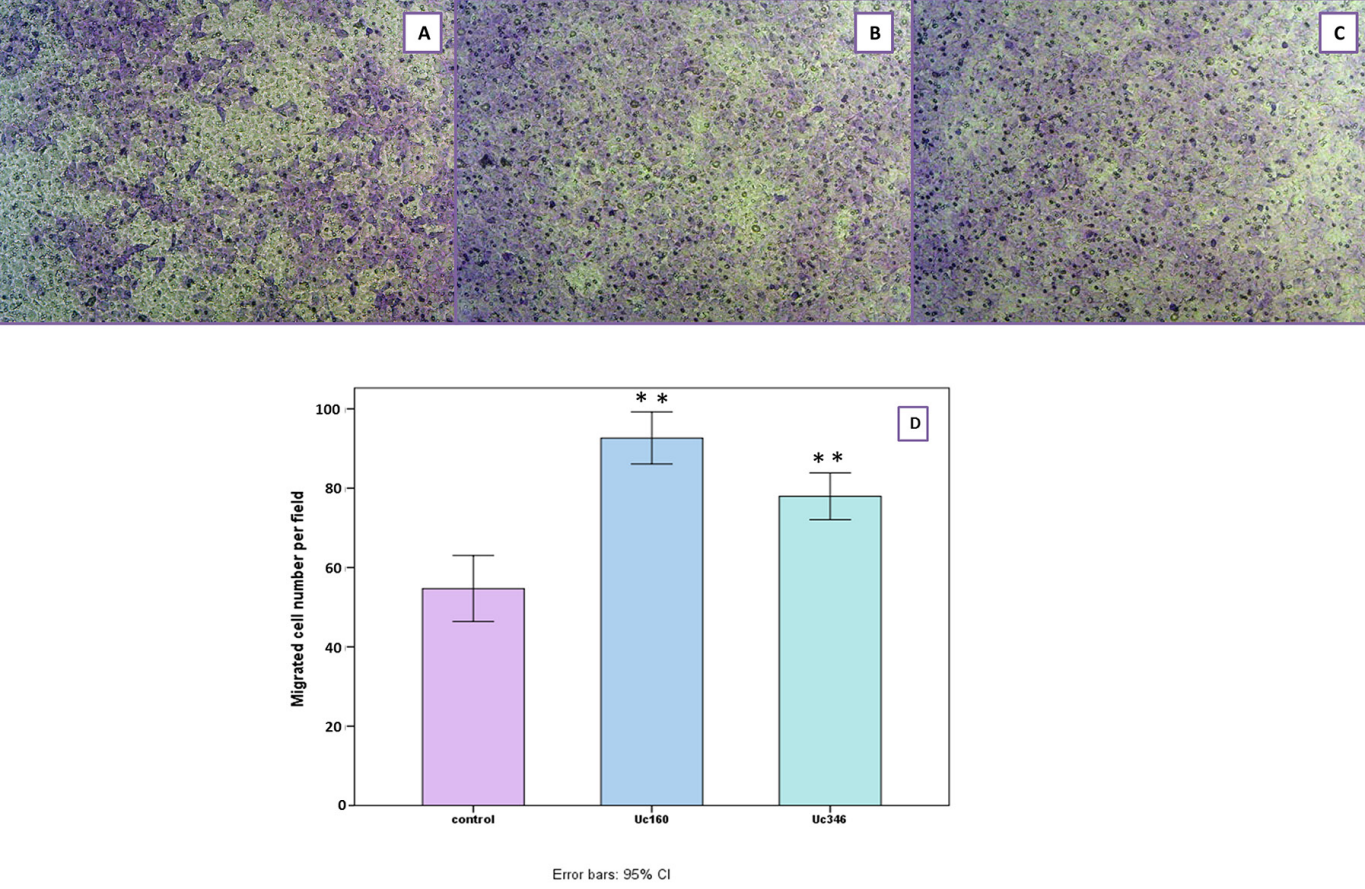
Migration of DLD-1 cells transiently transfected with Uc160 and Uc346 towards 10% FBS in comparison to control cells Representative images of control **(A)**, Uc160 **(B)** and Uc346 **(C)** at a magnification of 40x. Number of migrated cells per field **(D)** represented as mean ±SEM.

In the scratch wound healing assay, colon cancer cells that transiently overexpressed Uc160 or Uc346 displayed increased motility rates compared to the control at all time points and all cell lines (Figure 8). Although differences appeared small, the high reproducibility of the assay revealed that they were statistically significant. More specifically, Uc160 and Uc346 overexpressing HT-29 cells had increased motility rates at 24h compared to control cells (*p*=0.017 and *p*=0.041 respectively, Figure 8B and 8E), as well as DLD-1 cells (*p*=0.023 and *p*=0.004 respectively, Figure 8C and 8F). Differences in motility rates were also observed in Caco-2 cells at 24h, however without reaching statistical significance (*p*=0.260 and *p*=0.678 respectively, Figure 8A and 8D). Further analysis of DLD-1 cells with transwell migration assay confirmed the above results, with an increased number of Uc160 or Uc346 transfected cells migrating compared to control cells (*p*=0.005 for both T-UCRs overexpression) (Figure 9).

## DISCUSSION

UCRs have been associated with development and progression of many cancer types including CRC through aberrations in expression levels, methylation and even single nucleotide frequencies [28–40]. For example, expression levels of Uc73 and Uc388 in CRC are significantly decreased in tumor tissues while Uc73 levels have been correlated with overall survival [31]. Moreover, a cancer specific Uc160, Uc283 and Uc346 methylation pattern has been identified in colon cancer cells while DNA demethylation reversed T-UCR silencing [40].

In the present study, expression and methylation levels of Uc160, Uc283 and Uc346 were assessed in colorectal adenocarcinomas, paired non-malignant tumor-adjacent tissues and adenomas. Interestingly, we found that not only all CRC cases were methylated but also most normal tissues too. These findings provide a new insight on the presence of methylation in normal CRC tissue compared to previously reported findings whereby none of the normal colon tissues and only some of the colonic tumor tissue samples had methylated Uc160, Uc283 and Uc346 (72.29%, 65.85% and 46.91% respectively) [40]. This discrepancy in the results could be attributed possibly to differences in the sensitivity of the methodological approaches chosen (qMSP *vs* MSP) and the larger number of normal tissue samples used in our study (64 *vs* 5). Moreover, adenocarcinomas and adenomas exhibited higher levels compared to non-malignant tissues, in concordance with previous publications [40]. Surprisingly, methylation levels were highest in adenomas, probably due to the small number of adenoma tissue samples analyzed.

Furthermore, patients without metastasis displayed increased levels of Uc283 CRC tissue methylation compared to patients with metastasis. To our knowledge previous publications have focused only at lymph node and not distant metastases and reported a positive correlation between methylation status and positive lymph nodes for a pool of cancer patients with lymphoma, leukemia, colon, breast and lung cancer [40].

Since T-UCRs are highly methylated in CRC tissues compared to normal colon, higher levels of T-UCR expression were anticipated for non-malignant colon samples compared to CRC tissues. This was indeed observed for Uc160 and Uc283 (although not statistically significant for Uc283) but not for Uc346. This discrepancy may originate partly from the complex transcriptional regulation of T-UCRs that involves miRNA binding. Notably, Uc160 and Uc346 have been reported to have significant antisense complementarity to miRNAs, giving rise to six possible interacting pairs [28].

Another interesting observation was that b-actin levels were higher in CRC patients compared to healthy controls, adenoma patients or inflammation patients, reflecting the presence of a higher concentration of ccfDNA in accordance with previous publications, [7, 8, 41, 42]. Similarly, methylation levels of Uc160 and Uc346 were higher in the plasma of CRC patients compared to adenoma patients, inflammation patients or healthy controls as expected from the differences in methylation levels noted in the tissues. In addition, plasma Uc160 methylation levels were positively correlated with lymph node infiltration and lesion size (tumor or adenoma), possibly due to the higher levels of tumor DNA being released from the neoplastic lesion, which increases the detection sensitivity of ccfDNA, as has been observed for other molecules [43].

Along the same line, it is of great interest that none of the patients whose samples were collected postoperatively was positive for plasma Uc160 methylation, with the exception of a stage D patient without metastasectomy, reflecting active disease. Supportive to this observation is the case of a stage C patient who had positive plasma Uc160 methylation preoperatively and which turned to negative after the surgical removal of the tumor and the infiltrated lymph nodes. In accordance with our results, postoperative detection of other methylated markers such as serum *TFPI2* have been associated with R2 surgery, where active disease is present [44].

Of special interest is the negative plasma Uc160 methylation status observed in all 3 samples collected at different time points, preoperatively (from 2 different Departments) and postoperatively for a patient with CRC family history. This not only reinforces the reliability of the methodology but also raises the question of whether the role of T-UCRs differs between sporadic and familial CRC, since there was also a negative correlation between the methylation status of Uc160 and Uc346 and a family history of cancer. Such differences have been observed before in miRNA methylation levels, where higher methylation levels were noted in sporadic CRC cases compared to Lynch syndrome patients [45].

Considering the aforementioned findings, we explored the possibility of whether the methylation status of these three T-UCRs in plasma could constitute a minimally invasive biomarker for CRC or adenomas. Uc160 and the sum of the three T-UCRs were identified as the most promising biomarkers, with sensitivity and specificity 35% and 89%, and 45% and 74.3% respectively. So far, plasma methylation of *SEPT9* seems to have the highest sensitivity and specificity (reaching 95.6% and 84.8% respectively) for CRC, although these values drop to 9.6% for adenomas [46, 47]. Although these results led to the development of *SEPT9* plasma methylation kits for clinical use, retrospective studies reported conflicting results, with the sensitivity of the kit dropping to as low as 50.9% [46–49].

With regard to plasma methylation biomarkers for adenoma detection, the majority of published reports state relatively low specificities [50, 51]. Nevertheless there is an earlier study on plasma methylation of *RASSF2A*, *APC*, *MGMT* and *Wif-1* that suggested a more promising biomarker panel for adenoma with sensitivity of 86.5% and specificity of 92.1% [52]. It remains an interesting question whether this will be transferred to the clinic as a follow up study has not yet been published. In our study, inclusion of a larger cohort of adenoma patients could potentially uncover a similar role for Uc160 and Uc346 plasma methylation.

Since Uc160 and Uc346 seem to have a good potential as biomarkers, we aimed to assess their significance in tumor progression. Overexpression of Uc160 or Uc346 in CRC cell lines resulted to an increase in the proliferation as well as in the migration rate of the cells, supporting an oncogenic role for these long non-coding RNAs in the metastatic process of CRC, as it has been reported for other T-UCRs [53]. These findings are in line with the higher expression of Uc346 and lower methylation levels of Uc346 and Uc160 in CRC patients who tended to have distant metastasis more often compared to patients with lower Uc346 expression and higher methylation levels. It is of note that, although these T-UCRs are downregulated in CRC and CRC cell lines, overexpression led to increased proliferation and migration rate. Similar contrasting observations have been noted also for Uc73, whose upregulation associates with colon tumorigenesis [28]. On the contrary a positive correlation of Uc73 with overall survival was noted elsewhere, suggesting a potential role of Uc73 as a tumor suppressor in CRC [31]. These discrepancies further highlight the complicated role of T-UCRs in CRC evolution.

In conclusion, methylation and expression of Uc160, Uc283 and Uc346 are deregulated in CRC while Uc160 and Uc346 may participate in the metastatic process of CRC. Plasma methylation of Uc160, Uc283 and Uc346 is a promising biomarker for both CRC and adenomas mainly due to the good specificity observed. However, improvements in the methodological approach are necessary to increase the sensitivity of this assay.

## MATERIALS AND METHODS

**Figure 10:**
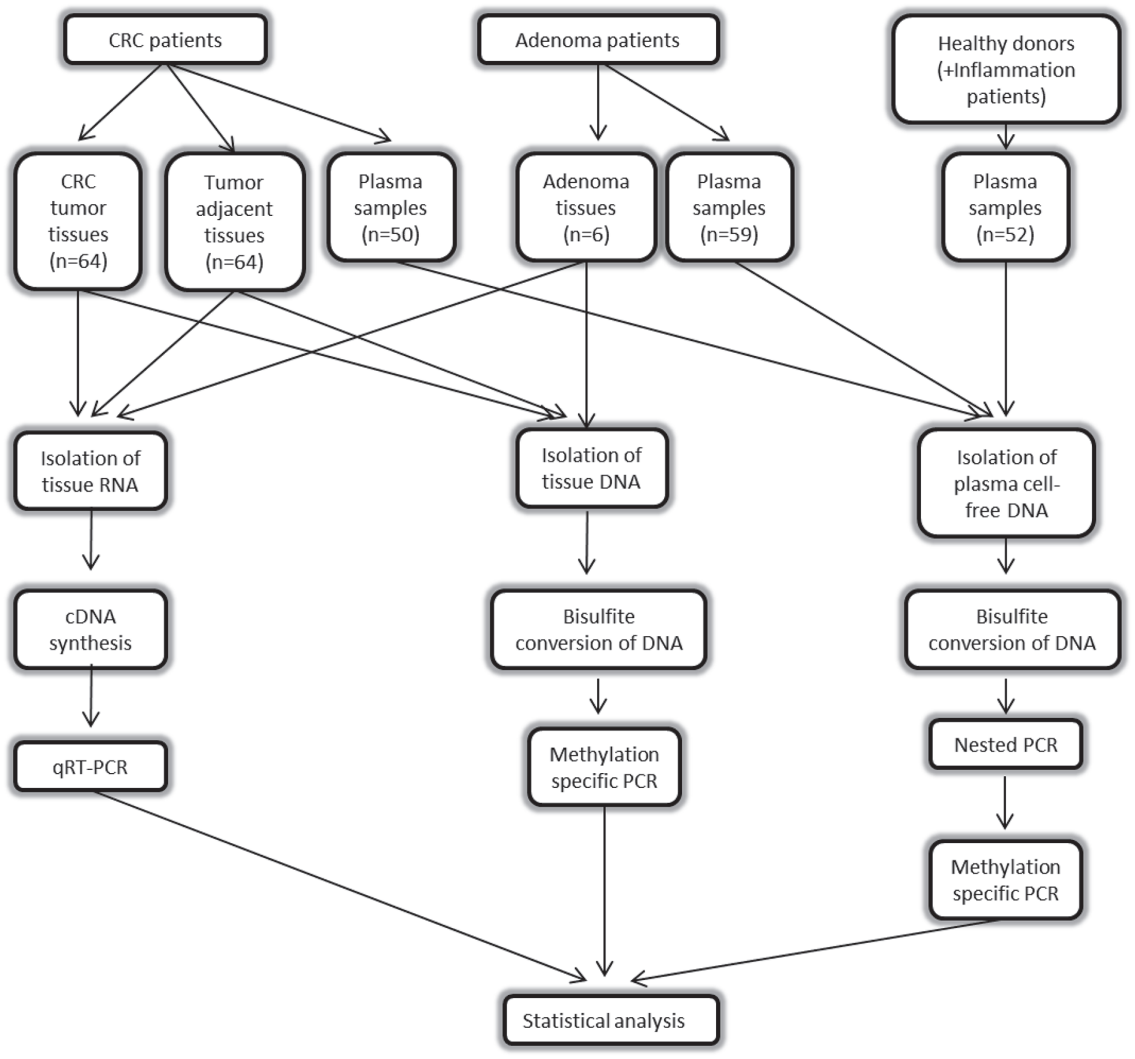
Workflow of the study

The outline of the workflow of our study is shown in Figure 10.

### Patients and samples

This study was approved by the local Research Ethics Committee of the University Hospital of Patras, Greece and informed consent was obtained from all participants. Sixty four patients with CRC, 59 patients with adenomas, 40 control subjects and 12 patients with colon inflammation were enrolled in the study at the University Hospital of Patras (Departments of Gastroenterology, Surgery and Medical Oncology) between November 2012 and April 2014. Clinical diagnosis was determined on the basis of colonoscopic findings and histological assessment. Control subjects underwent colonoscopy as part of a screening routine. Sixty four tumor and paired non-malignant specimens were obtained from the CRC patients and 6 adenoma specimens were obtained from the adenomas-only patients. Specimens were stored in *RNAlater* RNA Stabilization Reagent (Sigma–Aldrich, Austria) at −80°C. Blood samples were collected by phlebotomy prior to colonoscopy (Department of Gastroenterology) or prior to surgical excision of the tumor (Department of Surgery) or postoperatively (Department of Oncology) in K_2_EDTA Vacuette tubes (Greiner BioOne, Germany). Plasma was prepared within 2 hours of blood collection by centrifugation at 3000 rpm for 20 minutes at 4°C and was stored at −80°C until testing. Participants’ characteristics are shown in Table 7.

**Table 7:** Demographic and life style characteristics of the participants

Demographic characteristics		Cancer Patients	Adenoma Patients	Inflammation patients	Healthy individuals
Gender	Male	33 (66%)	38 (64.4%)	6 (50%)	12 (30%)
	Female	17 (34%)	21 (35.6%)	6 (50%)	28 (70%)
Age Group	60≥	16 (32%)	20 (33.9%)	4 (33.3%)	24 (60%)
	>60	34 (68%)	39 (66.1%)	8 (66.6%)	16 (40%)
Smoking	No	16 (32%)	30 (50.8%)	6 (50%)	32 (80%)
	Yes	9 (18%)	23 (39%)	5 (41.7%)	8 (20%)
Cancer Family History	No	7 (14%)	25 (42.4%)	5 (41.7%)	19 (47.5%)
	Yes	13 (26%)	26 (44.1%)	6 (50%)	17 (42.5%)
Coffee consumption	No	7 (14%)	9 (15.3%)	1 (8.3%)	6 (15%)
	Yes	13 (26%)	43 (72.9%)	10 (83.3%)	34 (85%)
Alcohol consumption	No	16 (32%)	26 (44.1%)	5 (41.7%)	22 (55%)
	Yes	8 (16%)	29 (49.1%)	6 (50%)	18 (45%)
Meat consumption	<2 times/wk	6 (3%)	9 (15.3%)	2 (16.7%)	7 (17.5%)
	>2 &<4/wk	3 (6%)	31 (52.5%)	3 (25%)	14 (35%)
	>4/wk	11 (22%)	11 (18.6%)	5 (41.7%)	18 (45%)
Exercise	Occasionally	13 (26%)	36 (61%)	7 (58.3%)	22 (55%)
	Often	11 (22%)	18 (30.5%)	4 (33.3%)	18 (45%)

### RNA preparation and cDNA synthesis

Total RNA was extracted from 80 mg neoplastic and paired non-malignant tumor adjacent FF tissue specimens from 51 CRC patients, as well as from 2 FF adenoma tissue specimens using the PerfectPure RNA Tissue Kit (5Prime, Hamburg, Germany), according to the manufacturers’ instructions. RNA samples were then incubated with DNase (Ambion, Austin, TX, USA) and quantified using a Nanodrop-1000 spectrophotometer (NanoDrop, Fisher Thermo, Wilmington, DE, USA). RNA integrity was checked by electrophoresis on 1% (w/v) agarose gels and the RNA was stored at −80°C. A total of 3 μg of RNA was reverse transcribed into cDNA using 100 U of Superscript III Reverse Transcriptase (Life Technologies), 300 ng of random primers (Foundation for Research and Technology-Hellas, Crete, Greece) and 5 nM dNTPs (Stratagene) in a total volume of 50 μl. A no enzyme control was used to ensure that the RNA samples were DNA-free. The mixture was incubated in a C1000 Touch thermal cycler (Bio-Rad) at 25°C for 5 minutes, 50°C for 60 minutes, and 70°C for 15 minutes. CDNA was diluted to 30 ng/μl and stored at −20°C.

### Quantification of T-UCRs expression

Expression levels of T-UCRs 160, 283 and 346 were quantified by pre-optimized real-time PCR (qPCR) assays (Primer-Design Ltd, Hants, UK) modified to allow the use of dual labeled hydrolysis probes (Supplementary Table 3). The qPCR reactions were carried out in triplicate in a total volume of 20 μl, containing 5μl of cDNA, 2×TaqMan Universal Master Mix II (Applied Biosystems, Foster City, CA) and 150 nM of the specific T-UCR primer mix. A no template control was used in all reactions. Cycling conditions were as follows: 95°C for 10 minutes, 45 cycles at 95°C for 15 seconds and at 60°C for 1 minute. Expressed Alu-Sq repeats were used as a reference gene, as it was validated to be a stably expressed repeat sequence in normal and tumor tissues of different grades and stages [54, 55]. Primers for the Alu-Sq were synthesized by Metabion International AG (Martinsried, Germany) [56]. The relative expression levels of each T-UCR were calculated using the LinReg Program [57] and were normalized to the Alu-Sq levels.

### DNA isolation and bisulfite conversion of tissue DNA

DNA was extracted from 25 mg neoplastic and paired non-malignant tumor adjacent FF tissue specimens from 64 CRC patients, as well as from 6 FF adenomas using the Nucleospin Tissue Kit (Macherey-Nagel, Düren, Germany) according to the manufacturer's instructions. DNA was eluted in 30 μl elution buffer. A total of 1 μg DNA was bisulfite converted and recovered using the Qiagen Epitect DNA bisulfite kit (Qiagen, Hilden, Germany). Bisulfite converted DNA was eluted in 40 μl elution buffer diluted in a total volume of 200 μl and stored at −20°C.

### DNA isolation and bisulfite conversion of ccfDNA

DNA was extracted from 4 ml plasma from 40 healthy participants, 12 patients with colon inflammation, 50 CRC patients and 59 adenoma patients using the QIAamp Circulating Nucleic Acid Kit (Qiagen, Hilden, Germany) according to the manufacturer's instructions. DNA was eluted in 50μl elution buffer. Sodium bisulfite conversion of DNA was performed by the EZ DNA Methylation-Gold Kit (Zymo Research Corporation, Orange, CA) according to the manufacturer's protocol. Bisulfite converted DNA was eluted in 30μl elution buffer and stored at −20°C.

### DNA methylation analysis of tissue and ccfDNA

The methylation status of T-UCRs 160, 283 and 346 in tissue and ccfDNA samples was detected by real-time quantitative Methylation Specific PCR (qMSP). Bisulfite treated-based methylation-specific primers and relevant Locked Nucleic Acid (LNA) dual labeled hydrolysis probes specific for methylated T-UCRs 160, 283 and 346 were designed using MethPrimer [58], following the NCBI library sequences (http://www.ncbi.nlm.nih.gov/). UCbase 2.0 (http://ucbase.unimore.it/) was also used for the identification of possible SNPs in Uc160, Uc283 and Uc346 promoter regions so that primers were designed outside regions with SNPs with known minor allele frequency of more than 0.01 [59] (Supplementary Table 4). Each primer and LNA probe was designed to bind to 2-3 CGs while assuring that no CGs were present in the region between the primers and the probe, so that the detected amplicons were fully methylated. Primers sequences were then subjected to BiSearch web-based primer analysis tool to ensure specificity of the bisulfite converted designed primers [60]. All primers and probes were synthesized by Metabion International AG (Martinsried, Germany) (sequences are shown in Supplementary Table 3). The qPCR reactions were carried out in triplicate, in a total volume of 20 μl, containing 5 μl of bisulfite converted DNA, 2× TaqMan Universal Master Mix II (Applied Biosystems, Foster City, CA), 300 nM of each primer and 75 nM of each probe. In all reactions three controls were used: a no template control, an unmethylated control (healthy donor blood DNA, bisulfite converted) and a fully methylated control (healthy donor blood DNA, *in vitro* methylated with SsI methylase, NEB Ipswich, MA and bisulfite converted). Serial dilutions of *in vitro* methylated DNA (100%, 10%, 1%, and 0.1%) were used to establish a standard curve. Methylation levels were finally normalized to β-actin levels, which was used as a reference gene, using previously reported primers and probe [61]. Cycling conditions were as follows: 95°C for 10 minutes and 50 cycles at 95°C for 15 seconds and at 60°C for 1 minute (Supplementary Table 3).

When analyzing ccfDNA samples, a first round of PCR was also performed for each T-UCR, with external primers that bind to CpGs-free bisulfite converted DNA. The qPCR reactions were carried out in a total volume of 20 μl, containing 6 μl of bisulfite converted DNA, 5x Mg-free KAPA Taq HotStart Buffer (Kapa Biosystems, Woburn, MA, USA), 400nM of each primer, 800 nM dNTPs, 1.25 nM MgCl_2_ and 1.5 U KAPA Taq HotStart DNA Polymerase (KAPABIOSYSTEMS, Woburn, MA, USA). Cycling conditions were 95°C for 3 minutes, 15 cycles at 95°C for 30 seconds, 56°C for 30 seconds and 72°C for 1 minute (see Supplementary Table 3 for detailed annealing temperatures). All samples were considered positive if at least one of the three replicates demonstrated amplification and depending on the amplification efficiency if the Cycle threshold (Ct) value for methylated Uc160 and Uc283 was ≤40, for methylated Uc346 ≤42 and for B-actin was ≤35.

Blind experimental design and analysis was performed with respect to the participants’ identities and data.

### Statistical analysis

IBM SPSS Statistics for Windows, Version 21.0 (Armonk, NY: IBMCorp) was used for all the analyses performed. Intergroup comparisons for the association of clinicopathological parameters with mRNA and methylation levels of Uc160, Uc283 and Uc346 were performed using Kruskal–Wallis and Mann–Whitney nonparametric tests. Comparisons between related groups were performed using Wilcoxon paired samples test for mRNA and methylation levels. Pearson correlation was used to detect potential correlations between T-UCRs methylation and mRNA levels, tumor size and methylation levels in plasma and tumors. Receiver Operating Characteristics (ROC) curve analysis and the area under the curve (AUC) were determined to define markers with highest sensitivity and specificity.

### Cell culture

Caco-2, HT-29 and DLD-1 colon cancer cell lines were purchased from the American Type Culture Collection (ATCC). DLD-1 and HT-29 cells were cultured in RPMI 1640 medium with 10% fetal bovine serum (FBS) and Caco-2 cells were cultured in Eagle's Minimum Essential medium (EMEM) with 10% FBS. Cells were cultured at 37°C, 5% CO2 and 100% humidity.

### Cloning and transfection

Lujambio et al. [40] showed that Uc160 and Uc346 are methylated in colon cancer cell lines. Therefore Uc160 and Uc346 were overexpressed in colon cancer cell lines and proliferation and migration were defined. Uc160 and Uc346 transcribed regions were amplified using two sets of primers for each T-UCR, one outer set and one inner set which included the restriction sites for XhoI and BamHI (Supplementary Table 5). PCR products as well as pcDNA3.1/Hygro(−) plasmid (Invitrogen) were digested with XhoI and BamHI, cleaned and ligated using DNA ligation kit (Takara). DH5a competent cells were transformed with the ligated products and plasmids were isolated from the cultures grown the selected colonies with Nucleospin Plasmid (Macherey – Nagel, Germany). Cell transfections were performed using TurboFect™ *in vitro* Transfection Reagent (Fermentas GmbH, Germany) for 20h according to the manufacturer's instructions. Transfection efficiency was confirmed before performing the proliferation and motility experiments (Supplementary Figure 1).

### Cell proliferation

The effect of Uc160 and Uc346 overexpression on proliferation of cells was determined using the 3-(4,5-dimethylthiazol-2-yl)-2,5-dephenyltetrazolium-bromide (MTT) assay. Briefly, Caco-2, HT-29 and DLD-1 cells were seeded at a density of 5 × 10^4^ cells/well in 24-well plates with full growth media and transiently transfected with 250 ng empty vector or plasmid with Uc160 or Uc346 for 20h. Cell proliferation was measured at 0h, 24h and 48h after transfection. MTT solution (5 mg/mL in PBS) was added to each well (1/10 of the volume) and incubated for 4 h at 37°C. Medium was removed and formazan crystals were solubilized in 100 μL acidified isopropanol. The solution was transferred to 96-well plates and read in a microplate reader (Tecan, Sunrise, Magellan 2) at a wavelength of 570 nm using reference wavelength 620 nm. Cell numbers were calculated using standard curves that were generated for each cell line using an increasing number of cells plated per well. The experiments were performed three times in triplicate.

### Scratch wound healing assay

Motility of Caco-2, HT-29 and DLD-1 cells was evaluated using the scratch wound healing assay. Briefly, cells were grown in 6-well plates in 90% confluency and transiently transfected with 1μg vector or plasmid with Uc160 or Uc346 for 20h. After transfection the cells were then wounded with a plastic pipette tip, rinsed with PBS and incubated with growth media. Images were captured at time 0h, 24h and 48h at a magnification of 5x using an Axiocam ERc 5s camera (Carl Zeiss Microscopy LLC) mounted on an inverted microscope (Axiovert 40 CFL, Carl Zeiss Microscopy LLC). The experiments were performed five times.

### Transwell migration assay

Cell migration of DLD-1 was further evaluated using the transwell migration assay. Cells were transiently transfected with Uc160 or Uc346 or vector for 20h and left in full growth medium post-transfectionally for 24h. Cells were suspended in serum-free culture medium and 1×10^5^ cells were loaded onto the top of Transwell chambers equipped with 8.0 μm pore-size polycarbonate membranes (Corning Inc., NY, USA) and allowed to migrate towards 10% FBS medium for 24h. Migrated cells were fixed with Carson's buffer and stained with Giemsa. Images were captured at a magnification of 40x using an Axiocam ERc 5s camera (Carl Zeiss Microscopy LLC) mounted on an inverted microscope (Axiovert 40 CFL, Carl Zeiss Microscopy LLC) and the average number of migrated cells was calculated using five different fields.

## SUPPLEMENTARY MATERIALS FIGURES AND TABLES



## Abbreviations

CRC: Colorectal cancer
UCRs: Ultraconserved regions
T-UCRs: Transcribed Ultraconserved regions
ccfDNA: circulating cell-free DNA
FF: fresh frozen
qPCR: quantitative PCR
qMSP: quantitative Methylation Specific PCR
LNA: Locked Nucleic Acid
MTT: 3-(4,5-dimethylthiazol-2-yl)-2,5-dephenyltetrazolium-bromide.

## References

[1] Siegel RL, Miller KD, Jemal A (2017). Cancer statistics, 2017. CA Cancer J Clin.

[2] Cress RD, Morris C, Ellison GL, Goodman MT (2006). Secular changes in colorectal cancer incidence by subsite, stage at diagnosis, and race/ethnicity, 1992-2001. Cancer.

[3] Siegel RL, Ward EM, Jemal A (2012). Trends in colorectal cancer incidence rates in the united states by tumor location and stage, 1992-2008. Cancer Epidemiol Biomarkers Prev.

[4] Levin B, Lieberman DA, McFarland B, Smith RA, Brooks D, Andrews KS, Dash C, Giardiello FM, Glick S, Levin TR, Pickhardt P, Rex DK, Thorson A (2008). Screening and surveillance for the early detection of colorectal cancer and adenomatous polyps, 2008: a joint guideline from the american cancer society, the us multi-society task force on colorectal cancer, and the american college of radiology. CA Cancer J Clin.

[5] Jahr S, Hentze H, Englisch S, Hardt D, Fackelmayer FO, Hesch RD, Knippers R (2001). DNA fragments in the blood plasma of cancer patients: quantitations and evidence for their origin from apoptotic and necrotic cells. Cancer Res.

[6] Rykova EY, Morozkin ES, Ponomaryova AA, Loseva EM, Zaporozhchenko IA, Cherdyntseva NV, Vlassov VV, Laktionov PP (2012). Cell-free and cell-bound circulating nucleic acid complexes: mechanisms of generation, concentration and content. Expert Opin Biol Ther.

[7] Perkins G, Yap TA, Pope L, Cassidy AM, Dukes JP, Riisnaes R, Massard C, Cassier PA, Miranda S, Clark J, Denholm KA, Thway K, Gonzalez De Castro D (2012). Multi-purpose utility of circulating plasma DNA testing in patients with advanced cancers. PLoS One.

[8] Schwarzenbach H, Hoon DS, Pantel K (2011). Cell-free nucleic acids as biomarkers in cancer patients. Nat Rev Cancer.

[9] Lavon I, Refael M, Zelikovitch B, Shalom E, Siegal T (2010). Serum DNA can define tumor-specific genetic and epigenetic markers in gliomas of various grades. Neuro Oncol.

[10] Weiss L, Hufnagl C, Greil R (2013). Circulating tumor DNA to monitor metastatic breast cancer. N Engl J Med.

[11] Tanaka H, Tsuda H, Nishimura S, Nomura H, Kataoka F, Chiyoda T, Tanaka K, Iguchi Y, Susumu N, Aoki D (2012). Role of circulating free alu DNA in endometrial cancer. Int J Gynecol Cancer.

[12] Zhou J, Shi YH, Fan J (2012). Circulating cell-free nucleic acids: promising biomarkers of hepatocellular carcinoma. Semin Oncol.

[13] Melnikov AA, Scholtens D, Talamonti MS, Bentrem DJ, Levenson VV (2009). Methylation profile of circulating plasma DNA in patients with pancreatic cancer. J Surg Oncol.

[14] Tomita H, Ichikawa D, Ikoma D, Sai S, Tani N, Ikoma H, Fujiwara H, Kikuchi S, Okamoto K, Ochiai T, Otsuji E (2007). Quantification of circulating plasma dna fragments as tumor markers in patients with esophageal cancer. Anticancer Res.

[15] da Silva Filho BF, Gurgel AP, Neto MA, de Azevedo DA, de Freitas AC, Silva Neto Jda C, Silva LA (2013). Circulating cell-free DNA in serum as a biomarker of colorectal cancer. J Clin Pathol.

[16] Fischer JR, Ohnmacht U, Rieger N, Zemaitis M, Stoffregen C, Manegold C, Lahm H (2007). Prognostic significance of RASSF1A promoter methylation on survival of non-small cell lung cancer patients treated with gemcitabine. Lung Cancer.

[17] Sunami E, Shinozaki M, Higano CS, Wollman R, Dorff TB, Tucker SJ, Martinez SR, Mizuno R, Singer FR, Hoon DS (2009). Multimarker circulating DNA assay for assessing blood of prostate cancer patients. Clin Chem.

[18] Mussolin L, Burnelli R, Pillon M, Carraro E, Farruggia P, Todesco A, Mascarin M, Rosolen A (2013). Plasma cell-free DNA in paediatric lymphomas. J Cancer.

[19] Schmidt K, Diehl F (2007). A blood-based dna test for colorectal cancer screening. Discov Med.

[20] Flamini E, Mercatali L, Nanni O, Calistri D, Nunziatini R, Zoli W, Rosetti P, Gardini N, Lattuneddu A, Verdecchia GM, Amadori D (2006). Free DNA and carcinoembryonic antigen serum levels: an important combination for diagnosis of colorectal cancer. Clin Cancer Res.

[21] Kin C, Kidess E, Poultsides GA, Visser BC, Jeffrey SS (2013). Colorectal cancer diagnostics: Biomarkers, cell-free DNA, circulating tumor cells and defining heterogeneous populations by single-cell analysis. Expert Rev Mol Diagn.

[22] Wallner M, Herbst A, Behrens A, Crispin A, Stieber P, Goke B, Lamerz R, Kolligs FT (2006). Methylation of serum DNA is an independent prognostic marker in colorectal cancer. Clin Cancer Res.

[23] Herbst A, Wallner M, Rahmig K, Stieber P, Crispin A, Lamerz R, Kolligs FT (2009). Methylation of helicase-like transcription factor in serum of patients with colorectal cancer is an independent predictor of disease recurrence. Eur J Gastroenterol Hepatol.

[24] Misale S, Yaeger R, Hobor S, Scala E, Janakiraman M, Liska D, Valtorta E, Schiavo R, Buscarino M, Siravegna G, Bencardino K, Cercek A, Chen CT (2012). Emergence of KRAS mutations and acquired resistance to anti-EGFR therapy in colorectal cancer. Nature.

[25] Bejerano G, Pheasant M, Makunin I, Stephen S, Kent WJ, Mattick JS, Haussler D (2004). Ultraconserved elements in the human genome. Science.

[26] Lowe CB, Bejerano G, Haussler D (2007). Thousands of human mobile element fragments undergo strong purifying selection near developmental genes. Proc Natl Acad Sci U S A.

[27] Katzman S, Kern AD, Bejerano G, Fewell G, Fulton L, Wilson RK, Salama SR, Haussler D (2007). Human genome ultraconserved elements are ultraselected. Science.

[28] Calin GA, Liu CG, Ferracin M, Hyslop T, Spizzo R, Sevignani C, Fabbri M, Cimmino A, Lee EJ, Wojcik SE, Shimizu M, Tili E, Rossi S (2007). Ultraconserved regions encoding ncRNAs are altered in human leukemias and carcinomas. Cancer Cell.

[29] Braconi C, Valeri N, Kogure T, Gasparini P, Huang N, Nuovo GJ, Terracciano L, Croce CM, Patel T (2011). Expression and functional role of a transcribed noncoding RNA with an ultraconserved element in hepatocellular carcinoma. Proc Natl Acad Sci U S A.

[30] Galasso M, Dama P, Previati M, Sandhu S, Palatini J, Coppola V, Warner S, Sana ME, Zanella R, Abujarour R, Desponts C, Teitell MA, Garzon R (2014). A large scale expression study associates uc.283-plus lncRNA with pluripotent stem cells and human glioma. Genome Med.

[31] Sana J, Hankeova S, Svoboda M, Kiss I, Vyzula R, Slaby O (2012). Expression levels of transcribed ultraconserved regions uc.73 and uc.388 are altered in colorectal cancer. Oncology.

[32] Hudson RS, Yi M, Volfovsky N, Prueitt RL, Esposito D, Volinia S, Liu CG, Schetter AJ, Van Roosbroeck K, Stephens RM, Calin GA, Croce CM, Ambs S (2013). Transcription signatures encoded by ultraconserved genomic regions in human prostate cancer. Mol Cancer.

[33] Scaruffi P (2011). The transcribed-ultraconserved regions: a novel class of long noncoding rnas involved in cancer susceptibility. Sci World J.

[34] Jiang J, Azevedo-Pouly AC, Redis RS, Lee EJ, Gusev Y, Allard D, Sutaria DS, Badawi M, Elgamal OA, Lerner MR, Brackett DJ, Calin GA, Schmittgen TD (2016). Globally increased ultraconserved noncoding RNA expression in pancreatic adenocarcinoma. Oncotarget.

[35] Fassan M, Dall'Olmo L, Galasso M, Braconi C, Pizzi M, Realdon S, Volinia S, Valeri N, Gasparini P, Baffa R, Souza RF, Vicentini C, D'Angelo E (2014). Transcribed ultraconserved noncoding RNAs (T-UCR) are involved in barrett's esophagus carcinogenesis. Oncotarget.

[36] Scaruffi P, Stigliani S, Moretti S, Coco S, De Vecchi C, Valdora F, Garaventa A, Bonassi S, Tonini GP (2009). Transcribed-ultra conserved region expression is associated with outcome in high-risk neuroblastoma. BMC Cancer.

[37] Lin M, Eng C, Hawk ET, Huang M, Greisinger AJ, Gu J, Ellis LM, Wu X, Lin J (2012). Genetic variants within ultraconserved elements and susceptibility to right- and left-sided colorectal adenocarcinoma. Carcinogenesis.

[38] Lin M, Eng C, Hawk ET, Huang M, Lin J, Gu J, Ellis LM, Wu X (2012). Identification of polymorphisms in ultraconserved elements associated with clinical outcomes in locally advanced colorectal adenocarcinoma. Cancer.

[39] Yang R, Frank B, Hemminki K, Bartram CR, Wappenschmidt B, Sutter C, Kiechle M, Bugert P, Schmutzler RK, Arnold N, Weber BH, Niederacher D, Meindl A (2008). SNPs in ultraconserved elements and familial breast cancer risk. Carcinogenesis.

[40] Lujambio A, Portela A, Liz J, Melo SA, Rossi S, Spizzo R, Croce CM, Calin GA, Esteller M (2010). CpG island hypermethylation-associated silencing of non-coding RNAs transcribed from ultraconserved regions in human cancer. Oncogene.

[41] Stroun M, Lyautey J, Lederrey C, Olson-Sand A, Anker P (2001). About the possible origin and mechanism of circulating DNA apoptosis and active DNA release. Clin Chim Acta.

[42] Herrera LJ, Raja S, Gooding WE, El-Hefnawy T, Kelly L, Luketich JD, Godfrey TE (2005). Quantitative analysis of circulating plasma DNA as a tumor marker in thoracic malignancies. Clin Chem.

[43] Philipp AB, Nagel D, Stieber P, Lamerz R, Thalhammer I, Herbst A, Kolligs FT (2014). Circulating cell-free methylated dna and lactate dehydrogenase release in colorectal cancer. BMC Cancer.

[44] Hibi K, Goto T, Shirahata A, Saito M, Kigawa G, Nemoto H, Sanada Y (2012). Methylation of tfpi2 no longer detected in the serum DNA of colorectal cancer patients after curative surgery. Anticancer Res.

[45] Pavicic W, Perkio E, Kaur S, Peltomaki P (2011). Altered methylation at microrna-associated CPG islands in hereditary and sporadic carcinomas: a methylation-specific multiplex ligation-dependent probe amplification (MS-MLPA)-based approach. Mol Med.

[46] Toth K, Sipos F, Kalmar A, Patai AV, Wichmann B, Stoehr R, Golcher H, Schellerer V, Tulassay Z, Molnar B (2012). Detection of methylated SEPT9 in plasma is a reliable screening method for both left- and right-sided colon cancers. PLoS One.

[47] Church TR, Wandell M, Lofton-Day C, Mongin SJ, Burger M, Payne SR, Castanos-Velez E, Blumenstein BA, Rosch T, Osborn N, Snover D, Day RW, Ransohoff DF (2014). Prospective evaluation of methylated SEPT9 in plasma for detection of asymptomatic colorectal cancer. Gut.

[48] Payne SR (2010). From discovery to the clinic: the novel dna methylation biomarker (m)SEPT9 for the detection of colorectal cancer in blood. Epigenomics.

[49] Warren JD, Xiong W, Bunker AM, Vaughn CP, Furtado LV, Roberts WL, Fang JC, Samowitz WS, Heichman KA (2011). Septin 9 methylated DNA is a sensitive and specific blood test for colorectal cancer. BMC Med.

[50] Cassinotti E, Melson J, Liggett T, Melnikov A, Yi Q, Replogle C, Mobarhan S, Boni L, Segato S, Levenson V (2012). DNA methylation patterns in blood of patients with colorectal cancer and adenomatous colorectal polyps. Int J Cancer.

[51] Zhang X, Song YF, Lu HN, Wang DP, Zhang XS, Huang SL, Sun BL, Huang ZG (2015). Combined detection of plasma GATA5 and SFRP2 methylation is a valid noninvasive biomarker for colorectal cancer and adenomas. World J Gastroenterol.

[52] Lee BB, Lee EJ, Jung EH, Chun HK, Chang DK, Song SY, Park J, Kim DH (2009). Aberrant methylation of APC, MGMT, RASSF2A, and Wif-1 genes in plasma as a biomarker for early detection of colorectal cancer. Clin Cancer Res.

[53] Wang C, Wang Z, Zhou J, Liu S, Wu C, Huang C, Ding Y (2017). TUC.338 promotes invasion and metastasis in colorectal cancer. Int J Cancer.

[54] Antonacopoulou AG, Grivas PD, Skarlas L, Kalofonos M, Scopa CD, Kalofonos HP (2008). POLR2F, ATP6V0A1 and PRNP expression in colorectal cancer: New molecules with prognostic significance?. Anticancer Res.

[55] Kottorou AE, Antonacopoulou AG, Dimitrakopoulos FI, Tsamandas AC, Scopa CD, Petsas T, Kalofonos HP (2012). Altered expression of NFY-C and RORA in colorectal adenocarcinomas. Acta Histochem.

[56] Rihani A, Van Maerken T, Pattyn F, Van Peer G, Beckers A, De Brouwer S, Kumps C, Mets E, Van der Meulen J, Rondou P, Leonelli C, Mestdagh P, Speleman F (2013). Effective alu repeat based RT-Qpcr normalization in cancer cell perturbation experiments. PLoS One.

[57] Ramakers C, Ruijter JM, Deprez RH, Moorman AF (2003). Assumption-free analysis of quantitative real-time polymerase chain reaction (PCR) data. Neurosci Lett.

[58] Li LC, Dahiya R (2002). Methprimer: designing primers for methylation PCRs. Bioinformatics.

[59] Lomonaco V, Martoglia R, Mandreoli F, Anderlucci L, Emmett W, Bicciato S, Taccioli C (2014). Ucbase 2.0: ultraconserved sequences database (2014 update). Database (Oxford).

[60] Aranyi T, Tusnady GE (2007). Bisearch: ePCR tool for native or bisulfite-treated genomic template. Methods Mol Biol.

[61] deVos T, Tetzner R, Model F, Weiss G, Schuster M, Distler J, Steiger KV, Grutzmann R, Pilarsky C, Habermann JK, Fleshner PR, Oubre BM, Day R (2009). Circulating methylated SEPT9 DNA in plasma is a biomarker for colorectal cancer. Clin Chem.

