# Supplementary material for: Deregulation of methylation of transcribed-ultra conserved regions in colorectal cancer and their value for detection of adenomas and adenocarcinomas
Source: Oncotarget. 2018 Apr 20;9(30):21411–28. doi: 10.18632/oncotarget.25115 (PMC5940382; doi:10.18632/oncotarget.25115)
Supplement: Supplementary file 1 [file oncotarget-09-21411-s001.pdf]

Deregulation of methylation of transcribed-ultra conserved regions in colorectal cancer and their value for detection of adenomas and adenocarcinomas

SUPPLEMENTARY MATERIALS

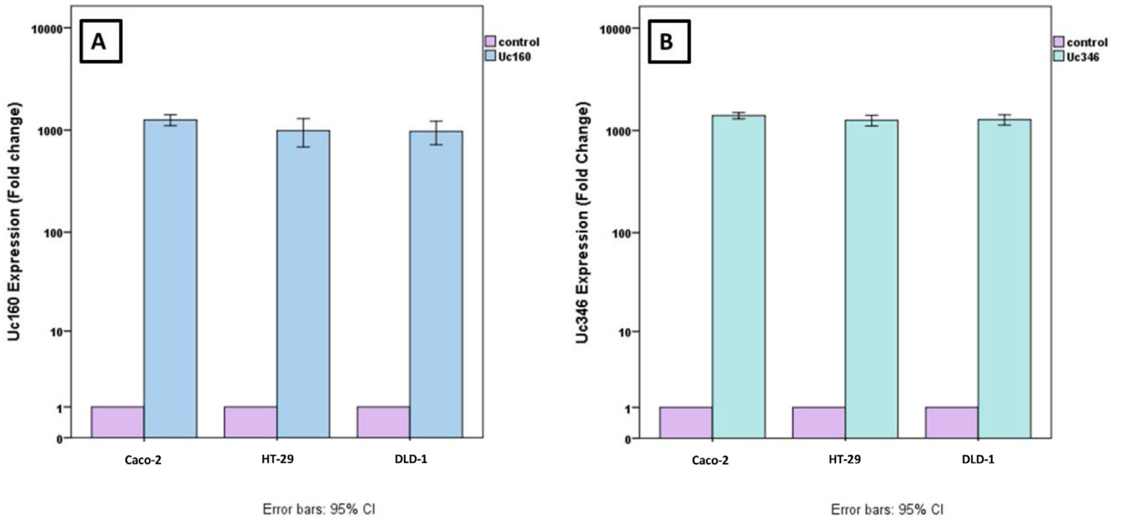

Supplementary Figure 1: Fold change expression of Uc160 **(A)** and Uc346 **(B)** upon transfection in Caco-2, HT-29 and DLD-1 colon cancer cells.

Supplementary Table 1: Clinicopathological parameters and relative RNA levels of Uc160, Uc283 and Uc346 in colorectal adenocarcinomas

Clinicopathological characteristics		Number of patients (%)	Uc160 relative expression	*P* value	Uc283 relative expression	*P* value	Uc346 relative expression	*P* value
Gender	Male	32 (62.7%)	0.57 (0.04-3.48)	0.459	0.45 (0-15.6)	0.601	0.11 (0-5.86)	0.335
	Female	19 (37.3%)	0.41 (0.05-1.7)		0.62 (0-5.61)		0.31 (0-2.65)	
Age Group	60≥	10 (19.6%)	0.63 (0.14-1.3)	0.38	0.57 (0.06-5.61)	1	0.13 (0-1.11)	0.706
	>60	41 (80.4%)	0.49 (0.04-3.5)		0.52 (0-15.6)		0.20 (0-5.86)	
Duke's Stage	A	3 (5.9%)	0.38 (0.14-.0.62)	0.248	2.87 (0.13-5.61)	0.887	0.78 (0-5.86)	0.366
	B	20 (39.2%)	0.75 (0.05-2.82)		0.62 (0-6.15)		0.16 (0-2.65)	
	C	20 (39.2%)	0.54 (0.04-3.48)		0.45 (0-15.63)		0.01 (0-1.81)	
	D	6 (11.8%)	0.16 (0.04-0.92)		0.94 (0.17-4.46)		0.31 (0-1.06)	
Grade	I	6 (11.8%)	0.21 (0.05-2.81)	0.410	0.52 (0.13-2.41)	0.696	0.75 (0-5.86)	0.667
	II	39 (76.5%)	0.66 (0.04-3.48)		0.70 (0-15.6)		0.16 (0-2.65)	
	III	1 (2%)	0.32		0.22		0.46	
Primary Site	Right Colon	21 (41.2%)	0.32 (0.04-1.59)	0.209	0.46 (0-6.15)	0.415	0.08 (0-1.06)	0.762
	Left Colon and Sigmoid	13 (25.5%)	0.66 (0.06-3.48)		0.70 (0-15.630		0.16 (0-5.86)	
	Rectum	15 (29.4%)	0.66 (0.04-2.81)		0.43 (0-5.61)		0.21 (0-2.65)	
Lymph Node metastasis	No	24 (47.1%)	0.62 (0.05-2.81)	0.328	0.56 (0-6.150	0.965	0.18 (0-5.86)	0.176
	Yes	25 (49%)	0.53 (0.04-3.48)		0.54 (0-15.63)		0.02 (0-1.81)	
Distant metastasis	No	39 (76.5%)	0.62 (0.04-3.48)	0.293	0.59 (0-15.63)	0.958	0.08 (0-5.860	0.160
	Yes	6 (11.8%)	0.37 (0.05-0.92)		0.66 (0.12-4.46)		0.45 (0-1.06)	

Supplementary Table 2: Clinicopathological parameters and relative DNA methylation of T-UCRs 160, 283 and 346 in colorectal adenocarcinomas

Clinicopathological characteristics		Number of patients (%)	Uc160 relative methylation	*P* value	Uc283 relative methylation	*P* value	Uc346 relative methylation	*P* value
Gender	Male	35 (54.7%)	0.71 (0-7.92)	0.846	0.27 (0-0.74)	0.973	0.01 (0-0.54)	0.586
	Female	29 (45.3%)	0.45 (0-11)		0.27 (0-2.34)		0 (0-1.83)	
Age Group	60≥	13 (20.3%)	1.45 (0-11)	0.06	0.28 (0.04-2.34)	0.707	0 (0-1.83)	0.764
	>60	51 (79.7%)	0.50 (0-8.11)		0.27 (0-1.09)		0.01 (0-1.78)	
Duke's Stage	A	3 (4.7%)	0.91 (0.26-1.57)	0.991	0.39 (0.28-0.740	0.205	0.14 (0-0.29)	0.896
	B	25 (39.1%)	0.82 (0-7.92)		0.25 (0-0.98)		0.01 (0-0.54)	
	C	26 (40.1%)	0.64 (0-11)		0.27 (0-1.85)		0 (0-1.78)	
	D	6 (9.4%)	0.47 (0-3.88)		0.16 (0.04-0.29)		0.01 (0-0.11)	
Grade	I	7 (10.9%)	1.4 (0-7.92)	0.807	0.43 (0-0.74)	0.052	0 (0-0.43)	0.249
	II	48 (78.5%)	0.59 (0-11)		0.25 (0.04-1.85)		0.01 (0-1.78)	
	III	1 (1.6%)	0.38		0		0	
Primary Site	Right Colon	27 (42.2%)	0.81 (0-11)	0.295	0.25 (0-1.85)	0.409	0.02 (0-0.92)	0.587
	Left Colon and Sigmoid	12 (18.8%)	1.45 (0-8.110		0.29 (0.06-1.090		0 (0-1.78)	
	Rectum	21 (32.8%)	0.35 (0-7.92)		0.21 (0-0.74)		0 (0-0.51)	
Lymph Node metastasis	No	29 (45.3%)	0.77 (0-7.92)	0.579	0.26 (0-0.980	0.520	0.01 (0-0.540	0.415
	Yes	31 (48.4%)	0.63 (0-11)		0.27 (0-1.86)		0 (0-1.78)	
Distant metastasis	No	50 (78.1)	0.82 (0-11)	0.390	0.26 (0-1.85)	0.021	0.01 (0-0.92)	0.152
	Yes	6 (9.4%)	0.34 (0-3.88)		0.06 (0-0.29)		0 (0.02)	

Supplementary Table 3: Primer and probe sequences and annealing temperatures for qRT-PCR, qMSP and nested PCR (+C: LNA nucleotide)

Primer/ Probe	Sequence (5'-3')	Annealing temperature (°C)
Expression assay (qRT-PCR) primers and probes		
Alu-sq FRW	CATGGTGAAACCCCGTCTCTA	57
Alu-sq REV	GCCTCAGCCTCCCGAGTAG	
Uc160 probe	ACTCCTTTCTGAACCAAACGGCAT	60
Uc283 probe	TTACAGAACCAATTATGCGCCATTAGACTTGCTT	60
Uc346 probe	AGGGCTGGGATTGCGTCGCTCTGA	60
Methylation assay (qMSP) primers and probes		
Uc160 FRW	ACGTTTATTCGGCGTC	60
Uc160 REV	CAACCCAAACTACGACC	
Uc160 probe	FAM/TTG +CGGTTT +CGTTTTA+CGA/BHQ1	
Uc283 FRW	ATTCGTTTTCGGGATTTGTTAG	63
Uc283 REV	CAAAACCACCGACTCCG	
Uc283 probe	FAM/T+CGTTTTTTT+CGGGT +CGGTTGTT/BHQ1	
Uc346 FRW	ACGGCGTTAGGGATTTCG	63
Uc346 REV	CGAATTACCCCGAATACTTTAACC	
Uc346 probe	FAM/TTT +CGTTTT +CGT +CG+CGGT T/BHQ1	
b-actin FRW	GTGATGGAGGAGGTTTAGTAAGTT	57
b-actin REV	CCAATAAAACCTACTCCTCCCTTAA	
b-actin probe	FAM/ACCACCACCCAACACACAATAACAAACACA/BHQ1	
Nested PCR primers		
Uc160 FRW	GAGGGATTTAAGTTTTTATTTTA	57
Uc160 REV	TCACCCTACCCAAATAC	
Uc283 FRW	GGTTTTTAGTTTTTTTGGTATG	56
Uc283 REV	AAAAAAAAATCACAAAAAACTTC	
Uc346 FRW	GGGGTTAGAGATTTTTATTT	56
Uc346 REV	CTATAATTAAAATATAAATAAAACCC	

Supplementary Table 4: T-UCRs names and positions according to Ucbase 2.0 (http://ucbase.unimore.it/)

Uc name	Chromosome	Start	End	Start hg18	End hg18	Upstream gene name	Downstream gene name
Uc160	5	77268844	77269165	77304600	77304921	AK128395	AP3B1
Uc283	10	50604757	50605033	50274763	50275039	AJ237663	ERCC6
Uc346	12	106976510	106976711	105500640	105500841	RPC2	RFX4

Supplementary Table 5: Primer sequences and annealing temperatures for Uc160 and Uc346 cloning (lower case for the sequence digested by the restriction enzymes)

Primer	Sequence (5'-3')	Product size (bp)	Annealing Temperature (°C)
Uc160 outer FRW	CCCTGCTCCTCGCCTTCC	439	65
Uc160 outer REV	CGCGCTCCCTCCAGGATG		
Uc160 inner FRW	*AA*ctcgagAGTAAATGAGGCGAGTGTG	322	65
Uc160 inner REV	*AA*ggatccAGCATCCTTAATATTTCCTTCC		
Uc346 outer FRW	CCCAGGCTCCAGGAGTTC	429	65
Uc346 outer REV	GCGGATGTTTCATGGGAAAAAG		
Uc346 inner FRW	*AA*ctcgagAAGGCTGGAGAAGGCCT	202	65
Uc346 inner REV	*AA*ggatccGGCCTGATGAATGGCCG		
